# RETRACTION: Effects of Dexmedetomidine on Surgical Site Wound Pain in Patients Undergoing Laparoscopic Cholecystectomy: A Meta‐Analysis

**DOI:** 10.1111/iwj.70298

**Published:** 2025-03-06

## Abstract

**RETRACTION**: WengJ. , ChengZ. , and LiS. , “Effects of Dexmedetomidine on Surgical Site Wound Pain in Patients Undergoing Laparoscopic Cholecystectomy: A Meta‐Analysis,” International Wound Journal 20, no. 10 (2023): 3657–3664, 10.1111/iwj.14256.37309086 PMC10588357

The above article, published online on 12 June 2023, in Wiley Online Library (http://onlinelibrary.wiley.com/), has been retracted by agreement between the journal Editor in Chief, Professor Keith Harding; and John Wiley & Sons Ltd. Following an investigation by the publisher, all parties have concluded that this article was accepted solely on the basis of a compromised peer review process. The editors have therefore decided to retract the article. The authors did not respond to our notice regarding the retraction.

**RETRACTION**: J. Weng , Z. Cheng , and S. Li , “Effects of Dexmedetomidine on Surgical Site Wound Pain in Patients Undergoing Laparoscopic Cholecystectomy: A Meta‐Analysis,” International Wound Journal 20, no. 10 (2023): 3657–3664, 10.1111/iwj.14256.37309086 PMC10588357

The above article, published online on 12 June 2023, in Wiley Online Library (http://onlinelibrary.wiley.com/), has been retracted by agreement between the journal Editor in Chief, Professor Keith Harding; and John Wiley & Sons Ltd. Following an investigation by the publisher, all parties have concluded that this article was accepted solely on the basis of a compromised peer review process. The editors have therefore decided to retract the article. The authors did not respond to our notice regarding the retraction.

